# Adapting preference-based utility measures to capture the impact of cancer treatment-related symptoms

**DOI:** 10.1007/s10198-021-01337-6

**Published:** 2021-06-17

**Authors:** Koonal K. Shah, Bryan Bennett, Andrew Lenny, Louise Longworth, John E. Brazier, Mark Oppe, A. Simon Pickard, James W. Shaw

**Affiliations:** 1 PHMR, London, UK; 2 Bristol-Myers Squibb, Uxbridge, UK; 3 School of Health and Related Research, University of Sheffield, Sheffield, UK; 4 Axentiva Solutions, Tenerife, Spain; 5 University of Illinois at Chicago, Chicago, IL USA; 6 Bristol-Myers Squibb, Lawrenceville, NJ USA

**Keywords:** Cancer, Oncology, Preference-based measure, Side effect, Adverse event

## Abstract

It is important that patient-reported outcome (PRO) measures used to assess cancer therapies adequately capture the benefits and risks experienced by patients, particularly when adverse event profiles differ across therapies. This study explores the case for augmenting preference-based utility measures to capture the impact of cancer treatment-related symptoms. Additional cancer treatment-related items could be specific (e.g., rash) or global. While specific items are easier to describe and understand, their use may miss rarer symptoms and those that are currently unknown but will arise from future medical advancements. The appropriate number of additional items, the independence of those items, and their impact on the psychometric properties of the core instrument require consideration. Alternatively, a global item could encompass all potential treatment-related symptoms, of any treatments for any disease. However, such an item may not be well understood by general public respondents in valuation exercises. Further challenges include the decision about whether to generate de novo value sets for the modified instrument or to map to existing tariffs. The fluctuating and transient nature of treatment-related symptoms may be inconsistent with the methods used in conventional valuation exercises. Fluctuating symptoms could be missed by sub-optimal measure administration timing. The addition of items also poses double-counting risks. In summary, the addition of treatment-related symptom items could increase the sensitivity of existing utility measures to capture known and unknown treatment effects in oncology, while retaining the core domains. However, more research is needed to investigate the challenges, particularly regarding valuation.

## Introduction

For many years, chemotherapy, surgery and radiotherapy have been the most common forms of cancer treatment available. More recently, dramatic improvements have been made in the field of immunotherapy, which has become an important therapeutic alternative and is now the first choice in many cases [1]. Immunotherapy enables the immune system to fight against cancer, infections, and other diseases. It has been shown to be effective in treating a range of advanced and metastatic cancers [2]. Recent successes have spurred a rapid increase in the number of immuno-oncology therapies being developed [3]. Traditional therapies for cancer, including chemotherapy and radiotherapy, are in general poorly tolerated, being associated with a plethora of (often severe) toxicities ranging from hair loss to bruising and bleeding. The advent of immuno-oncology has led to its widespread adoption as a new standard of care for multiple tumour types, thanks not only to its efficacy but also its tolerability relative to conventional treatments. For example, a recent meta-analysis of 22 randomized clinical trials involving 12,727 patients with solid organ malignancies concluded that patients receiving immunotherapy were less likely to develop severe treatment-related symptoms (also referred to as side effects, adverse effects, adverse events and treatment risks) than those receiving traditional chemotherapy [4]. Nevertheless, as experience with immuno-oncology has grown, concerns have arisen regarding certain treatment-related symptoms, including fatigue, diarrhoea, nausea and respiratory problems [5, 6]. Since candidate treatments tend to differ in terms of the severity of these effects and patients’ ability to tolerate them, it is important that any patient-reported outcome (PRO) measures used to assess the impact of treatments are able to adequately capture both their positive and negative effects [7].

The aim of this commentary paper is to examine the adequacy of existing generic and condition-specific preference-based measures for capturing important outcomes in cancer, and to explore the case for modifying or adding items to existing measures to capture the impact of treatment-related symptoms. This commentary paper provides a targeted overview and discussion of the literature and current issues, with the view to encouraging further discussion within the health economics and outcomes research field, and to informing future PRO development and refinement in the field of oncology.

The paper is structured as follows. First, the types of PRO measures used in cancer are described. The suitability of descriptive systems for capturing health effects of treatment is then discussed, with reflections on how existing generic measures could be adapted, and on how condition-specific measures have dealt with the issue. Next, challenges relating to valuation, capturing transient events, and modeling are explored. Finally, future steps towards addressing these challenges are summarized.

### Generic and condition-specific preference-based measures used in cancer

PROs can be delineated in a variety of ways as measures of health/health-related quality of life, notably as generic or condition-specific measures, and as preference-based or non-preference-based measures (note that the term ‘preference-based measure’ has been criticized as these measures are also used in applications where social preference weights are not relevant; ‘preference-accompanied measure’ has been suggested as an alternative [8]). There has been tremendous interest in preference-based measures in recent years due to their relevance in economic evaluations, as they can facilitate the calculation of quality-adjusted life years [9].

The EQ-5D, Health Utilities Index Mark 3 (HUI3), and SF-6D are three of the most prominent generic preference-based measures. A review of the psychometric properties of these instruments by Longworth et al. [10] showed that of the three, EQ-5D was by far the most commonly used in oncology, with 71 of the 98 studies reviewed reporting EQ-5D utility data (compared to 24 and three studies reporting HUI3 and SF-6D data, respectively). While there is evidence that EQ-5D is valid and reliable in many cancers [10, 11], there are concerns that this and other such generic measures are not sensitive enough and inevitably miss domains that are important in capturing the benefits and risks of new cancer treatments [12, 13].

Condition-specific measures are, therefore, preferred in some situations because by focusing on the condition of interest, they cover important dimensions that generic measures may miss, and can be more sensitive for a given dimension [14]. It is important to note that generic and disease-targeted measures are often used for different purposes. In cancer, examples of condition-specific measures include the European Organization for Research and Treatment of Cancer Quality of Life Questionnaires (EORTC QLQ) [15], the MD Anderson Symptom Inventory (MDASI) suite of measures [16] and the Functional Assessment of Cancer Therapy (FACT) family of instruments [17]. Since these measures have been developed specifically for use in cancer, they tend to offer greater content validity than generic measures within the oncology setting [18], when used for the intended purposes. On the other hand, for the purpose of serving as a health status descriptor relevant for evaluation in the general population, the content validity of QLQ-C30/FACT-G (and, therefore, QLU/FACT-8D) can be questioned.

However, the use of condition-specific measures poses problems in achieving cross-program comparability [19]. Many cancer-specific measures are not preference based or amenable to valuation. This means that they cannot be used to calculate quality-adjusted life years (QALYs), thereby precluding their use in cost-utility analysis. As an alternative to developing an entirely new cancer-specific preference-based measure, researchers have developed mapping functions that allow the conversion of outcomes from a non-preference-based measure to the values for a preference-based measure [10, 20]. The ISPOR Good Practices Task Force Report on mapping to estimate health-state utility from non-preference-based outcome measures provides methodological recommendations to analysts undertaking such studies [21]. Further recommendations on best practices for reporting the results of utility mapping studies have been provided in the MAPS (MApping onto Preference-based measures reporting Standards) statement [22]. However, mapping is unsuitable in situations where there is little overlap in content between the two measures, and it should not be used when the target preference-based measure is considered inappropriate.

Another approach is to take existing non-preference-based measures and reduce them so as to make them amenable to valuation [23]. This usually involves using psychometric criteria to select a subset of items from the existing measure and to analyze the performance of the candidate items. Relevant methods include factor analysis (a technique for identifying structurally independent dimensions with low correlation between each other), Rasch analysis (a technique that uses logistic models to convert categorical responses to points on a continuous scale), and assessments of validity and responsiveness. For example, the EORTC Quality of Life Utility Measure (EORTC QLU-C10D) is a health state classification system based on the larger EORTC QLQ-Core 30 (C30) cancer-specific quality of life questionnaire [24]. The QLU-C10D, which succeeded the EORTC 8D [25], comprises 10 dimensions, linked to 13 items selected from the 30 items of the QLQ-C30. The QLU-C10D has been valued using discrete choice experiments, and several national value sets have been reported [26–29]. The QLU-C10D valuation studies included a duration attribute to enable the anchoring of values onto the QALY scale [30]. Similarly, the FACT-8D is an eight-dimension preference-based measure derived from the FACT–General (FACT-G) questionnaire [31]. However, this approach may not be sufficient as many existing non-preference-based based measures such as the EORTC QLQ-C30 and the FACT-G were developed when chemotherapy was the dominant treatment paradigm in oncology. Since this time, the treatment landscape has evolved significantly and, therefore, many items in these measures may not be fully valid.

Yet even preference-based condition-specific measures may be problematic as they involve naming the condition (which can lead to bias [32]), lack a common upper anchor, and often miss impact on possible co-morbidities [33]. Valuation study respondents may exaggerate the importance of problems associated with the condition underpinning the health states under evaluation compared to other conditions (thereby leading to relatively large utility decrements) due to the psychological tendency to focus on what is placed in front of them [19], though this finding has not always been observed in the QLU-C10D valuation studies [26–29]. These concerns may undermine consistency in making comparisons between QALYs calculated using different measures. While some measures include domains representing known treatment-related symptoms (the QLU-C10D includes fatigue, appetite and nausea dimensions, for example [24]), unknown and less common side effects tend to be missed. Looking to the future, the effects of emerging innovative oncology treatments may be different from those of the chemotherapeutic regimens of past eras or from current immunotherapy options, and the cancer-specific measures previously developed may no longer be well suited to capture the array of health impacts.

Health technology assessment (HTA) agencies requiring cost-utility analyses generally prefer generic measures over condition-specific measures to promote consistency and comparability across appraisals [34]. However, preference-based condition-specific measures are sometimes accepted by HTA agencies in cases where there is evidence that the use of a generic measure is inappropriate, e.g., due to poor psychometric performance in the relevant patient group [35]. For example, in a National Institute for Health and Care Excellence (NICE) appraisal of fluocinolone acetonide intravitreal implant for the treatment of chronic diabetic macular oedema, the manufacturer collected quality of life data using a vision-specific questionnaire, the NEI-VFQ-25 [36]. NICE’s appraisal committee accepted that a disease-specific instrument was likely to be more responsive to changes of relevance to patients than the Institute’s preferred generic measure, the EQ‑5D.

### Adaptation of existing measures

While it is common to include both generic and condition-specific measures in a clinical trial data collection strategy (indeed, some generic measures have been designed to be used alongside other, more detailed measures) [37], it is often desirable to limit the number of instruments in a given study to reduce patient, investigator and operational burden. A potential compromise is to develop an adapted version of a generic measure for use in specific diseases. This notion has parallels with the extension of condition-specific measures for use in specific subtypes of the disease. For example, the FACT-G is considered appropriate for use in patients with any form of cancer, and is complemented by variants that include questions specific to particular sites/tumors (e.g., FACT-C for colorectal cancer) [38, 39]. The EORTC QLQ and MDASI instrument groups also have modules covering symptoms relevant for specific patient populations, intended to complement the core questionnaires or items.

One way of adapting a generic measure is by modifying the descriptive system to include additional dimensions of health. In the context of the EQ-5D, such dimensions have been described as ‘bolt-on’ items. Such an approach could improve the performance of the measure in certain settings, whilst retaining the general structure and conceptual framework underpinning the original measure and achieving better consistency with any utility values associated with the original measure. Existing research has examined the impact of expanding the EQ-5D to include bolt-on dimensions such as cognition [40], psoriasis (skin irritation and self-confidence) [41], sleep [42], vision, hearing, tiredness [43] and respiratory problems [44], amongst others. Beyond the EQ-5D, Brazier et al. have examined the impact of adding a pain and discomfort dimension to the AQL-5D, an asthma-specific preference-based measure [45]. Cancer has been a key area in which preference-based approaches have been applied to disease-specific measures, thereby offering some insight into opportunities for bolt-ons [46].

**Fig. 1:**
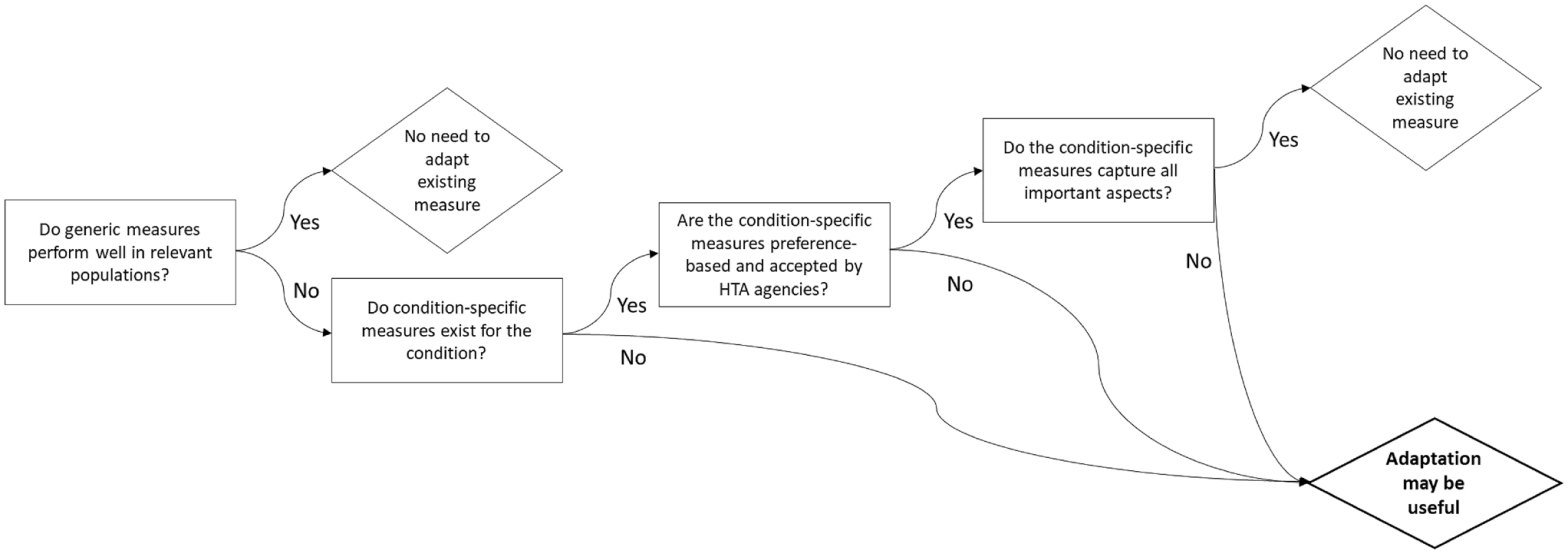
Questions to consider when determining the case for adapting an existing generic measure

Figure 1 shows when the adaptation of an existing generic measure may be justified—namely, if the generic measures fail to pick up important aspects of health and show poor psychometric properties in the relevant patient populations, and if measures specific to the condition of interest either do not exist or are otherwise inadequate [47]. It should be noted that these are necessary but not sufficient conditions for adapting a measure. Ultimately, the adaptation should improve the psychometric properties of the measure, i.e., it should address existing concerns about its content or face validity amongst the relevant patient population, and should matter to people to the extent that it would make a difference to utility values (though there are challenges involved in assessing this; see below) and ultimately to cost-effectiveness estimates. Psychometric methods such as principal component analysis can be used to identify gaps and identify candidate bolt-on dimensions for measures [48]. Principal component analysis involves examining a matrix of item correlations to reduce the information into a smaller set of components, with high intercorrelations implying that items are measuring the same latent component. Components can then be selected based on their eigenvalues, which represent the relative share of total variance accounted for by each component [49].

### Capturing treatment-related symptoms

The QLU-C10D comprises multiple concepts, including items relating to functioning (physical, role, social and emotional) and disease-related symptoms such as pain. It also includes items that capture common side effects of cancer treatments, such as nausea and bowel problems. However, it lacks a general (or ‘global’) treatment-related symptoms item. According to King et al. [24], this reflects the convention that attributes in utility instruments typically represent specific domains of health. By contrast, amongst the FACT measures, both the general and more specific questionnaires contain a global side effects item (FACIT GP5) which asks respondents to indicate the extent to which they are ‘bothered by side effects of treatment’ using a five-point scale. This is consistent with the US Food and Drug Administration recommendation that the adverse consequences of treatment are measured separately from treatment effectiveness [50].

The absence of a global side effects item means that measures such as the QLU-C10D may miss the full range of possible treatment-related symptoms, including for example, immune-related side effects such as breathing problems, rash and impacts on physical appearance [6] that do not correspond to any of the measure’s existing items (though shortness of breath is included in the larger QLQ-C30 questionnaire). It is practically difficult to identify an encompassing set of symptoms when using specific items rather than a global item [51]. Scientific understanding of immune-related side effects is evolving as novel classes of immuno-oncology therapies come to market. As experience with these agents grows, it is plausible that further important treatment-related symptoms may be identified in the future that are not well captured by existing items in these measures.

As noted above, an alternative to such cancer-specific measures would be to use a generic preference-based measure and to add items designed to improve its performance in oncology. Treatment-related symptoms could be captured via a global item or one or more specific items. A global item would allow the capturing of all possible side effects, including those that are less common or not currently known. This could allow researchers to more effectively compare new treatments versus standard of care by adding information on the severity of their respective side effect profiles. However, it may be difficult to frame a global treatment-related symptoms item in a way that reflects how patients themselves think about and describe their health and treatment. Patients may not use terms like ‘treatment-related symptoms’ (though phrases such as ‘bothered by the effects of your treatment’ may overcome this concern), and indeed may not know whether a particular health problem they are experiencing is a symptom of their disease or a consequence of their treatment. In other disease areas, single-item ratings of side effects have not been recommended due to concerns about their lack of reliability and sensitivity to change [52]. On the other hand, items describing specific side effects, such as breathing problems, are likely to be better understood, but adding only one or two items may be insufficient given the large variety of symptoms associated with cancer therapy in practice. Adding a large number of items may be undesirable as this introduces the risk that the brevity and core structure of the original instrument will be lost, i.e., the more dimensions that are added, the more likely it will be that the additional dimensions double-count the same construct (double-counting is also a concern for the global item approach due to overlap between the perceived adverse effects of treatment and impacts on core domains, particularly domains related to discomfort). A key challenge is to find a balance between the two competing options to describe side effects.

### Valuation issues

Condition-specific measures can in principle be valued using stated preference methods, as demonstrated by the recently published suite of QLU-C10D value sets [26–29]. However, if a generic measure is preferred, then adding items to existing measures may overcome this problem by placing the condition-specific element within the context of a broader health status measure, thereby potentially lessening the impact of focusing effects. This would necessitate the generation of a new value set for the augmented measure [44]. Not only would this be a very expensive process, but the new value set could be discordant with the existing value sets, e.g., the rank order of existing parameters could change. While the possibility of such findings should not deter research in this area, it would undoubtedly introduce challenges for HTA agencies who may be faced with possible ‘gaming’ due to the choice of multiple value sets, each with different properties. A potential solution has been suggested by Yang et al. [53], who explored the feasibility of using parameters from existing EQ-5D-5L value sets to predict values when new items are added. These were used as fixed parameters in modeling the bolt-on data, with a scale parameter introduced to capture the effects of adding the bolt-on item. The new items are valued as an additive or multiplicative deviation from the existing tariff. However, the evidence base supporting this approach is limited, and complications may arise if the new items interact with and affect the relative weightings of the existing items.

Further, health state valuations are conventionally derived from the preferences of the general population [54], as opposed to current patients. It is not clear whether a global item describing treatment-related symptoms would be understood by such individuals, particularly if they have never before experienced an unexpected adverse effect of treatment. Lack of familiarity with treatment burden may have contributed to general population samples placing relatively low weighting on specific symptom items in the QLU-C10D. A vignette valuation approach may help to provide the necessary context, though this is associated with other limitations such as inflexibility and challenges in incorporating into economic models [14].

A further issue in valuation relates to possible bias and focusing effects when specifying that symptoms are related to treatment. Valuation survey respondents may place a different amount of emphasis on symptoms if they are told that these are caused by treatment rather than by the disease, even if the impact of the symptoms on patients’ health and lives is the same irrespective of their cause. This may be an argument for favoring specific items that are not framed as being treatment related.

### Challenges of implementation: capturing transient events

Treatment side effects may be impactful, but are often short lived or variable over the course of treatment. Such fluctuations in health pose measurement challenges. Sanghera and Coast [55] note that when health fluctuates, standard measurement and analytic approaches may not be suitable due to recall periods and the timing of assessment. This is due to a phenomenon known as ‘recall bias’ in which the length of recall periods can introduce error or bias into clinical trials. For example, if the period is too long, it may lead to cognitive distortion in memory of an event (e.g., an event being perceived as less severe as when it was experienced); if too short, it may not allow enough time for an outcome to occur [56, 57].

The EQ-5D asks respondents to self-report their health status ‘today’, so the health state reported could differ depending on whether or not the symptoms of treatment are being experienced on the day of questionnaire completion (though this also applies to the core dimensions, and can be addressed by optimizing the timing of measurement; see below). Questionnaires with longer recall periods may run into other issues (such as the FACT-G which asks about the ‘past seven days’) since it is unclear whether respondents should consider their average health or worst health experiences over that period [17]. The QLQ-C30 mixes recall periods, with some items framed in the present tense (e.g., ‘Do you have any trouble […]’) and other items—including those covering common side effects—covering a one-week recall period [15].

Fixed-duration recall periods may be problematic in the context of health state valuation, particularly using techniques such as time trade-off which posit that the health state in question is experienced for a specified duration that differs from the measurement recall period (conventionally 10 years in many valuation protocols [58]). For this reason, when valuing the preference-based QLU-C10, all 10 dimensions are framed in the present tense, in contrast to the corresponding QLQ-C30 items. The use of fixed health state durations, like 10 years, in valuation may be problematic for side effects and other episodic or intermittent changes in health, irrespective of the recall period used in the measure. EQ-5D valuation studies, for example, require valuation survey respondents to imagine that they will experience the specified health problems (e.g., moderate problems in walking about) for 10 years, with no variation in the level of those problems throughout that period [58]. Although some respondents may question whether such a scenario is realistic, it is at least straightforward to specify and comprehend. It is less clear how a health state involving occasional or fluctuating levels of problems with treatment-related symptoms (or indeed fluctuating disease symptoms) would be described over a 10-year period. Some researchers have attempted to find solutions for valuing profiles in which health varies over time [59, 60]. An issue encountered is that respondents tend to neglect information about the amount of time spent in symptomatic states.

Related to recall period is the issue of timing of assessment. Patients may or may not be experiencing side effects at the point of questionnaire completion (which suggests that longer recall periods, more frequent collection, or event-driven questionnaire completion may be appropriate). The side effects of certain cancer treatments may be predictable. For example, if the adverse effects of chemotherapy typically occur during the first week of treatment and recede by the next administration of treatment, then measurement on the day of treatment would miss the impact of these side effects [55]. To capture fully the impact of side effects—whether via a bolt-on dimension or not—it is important to optimize the timing of measurement in clinical trials to reflect fluctuation patterns that are known and predictable [50]. Advances in the electronic collection of PRO data are expected to facilitate greater flexibility in this regard, allowing patients to self-report their health status at time points that are relevant, and not merely operationally convenient. If it is possible to capture PROs when symptoms occur, this would lessen the recall bias associated with retrospective data collection.

### Challenges of implementation: modeling

If important side effects are omitted from a given PRO measure, and therefore from the health state utility values associated with that measure, analysts may adjust the utility data to capture the impact of these side effects in economic models. Indeed, the ISPOR Good Practices Task Force Report on the identification, review, and use of health state utilities in cost-effectiveness models [61] explicitly recommends assessing “the extent to which the utility effects of important adverse events are captured by the data used to estimate a model’s non-adverse-event HSUs [health state utilities]” (p.273).

In practice, disutility values relating to treatment-related symptoms are typically sought from the literature and applied by subtracting the disutility from the utility value associated with the health state of interest or multiplying a weighting associated with the adverse event with the value of the health state of interest. These approaches risk double-counting if the main utility values already partially reflect the impact of those symptoms because the measure used captures them implicitly (this kind of double-counting issues is likely to occur when using any measure that describes symptoms). Further, the disutility values are often sourced or synthesized from data from multiple studies, which may be of variable methodological quality that used different, non-comparable valuation methods, and may not all have examined exactly the same side effect as the one being incorporated in the model. In some cases, disutility values for side effects are omitted from the model due to the lack of relevant data [62].

The inclusion of specific treatment-related symptoms (core or additional) items could help mitigate these issues. Notwithstanding the valuation issues described above, the valuation of the treatment-related symptoms would be combined with the valuation of the other health outcomes, thereby ensuring consistency in the methods used to generate the utility data. However, in order for such an item to demonstrate useful psychometric properties, the framing of the items and the frequency and timing of the data collection would need to be optimized so that the (sometimes transient) symptoms are not systematically missed at the point of questionnaire completion. It would also need to be demonstrated that the incidence rates of these side effects are sufficiently high, and their expected impact on quality-adjusted life years is sufficiently great, so as to justify their inclusion in the measure.

### Limitations

This commentary paper does not present any data or analyses that could be used to examine empirically some of the conjectures and discussion points presented. The points raised were drawn from the literature and the authors’ own knowledge and experiences, but no systematic review of the literature was undertaken. We are not aware of any existing reviews of studies to augment preference-based measures in general, but refer readers to a review of studies of bolt-ons specifically for the EQ-5D [63]. This commentary paper has focused on oncology, largely due to the importance of treatment-related symptoms when assessing and comparing immuno-oncology therapies. Some of the points raised may not be generalizable to other disease areas. However, the schematic shown in Fig. 1 is not specific to oncology and can be applied to any condition. Questions such as whether a global or specific treatment-related symptoms item is preferred are relevant in disease areas beyond oncology. For example, in systematic lupus erythematosus, researchers responsible for developing the LupusPRO opted to include items describing specific treatment-related symptoms as well as a general item capturing ‘bothersome side effects’ [64].

## Conclusions

When a preference-based measure of health is required, an additional layer of complexity is cast upon the acknowledged strengths and limitations of generic and disease-specific measures. Adapting existing generic preference-based measures by adding treatment-related symptoms items potentially improves their sensitivity to health-related changes/differences in cancer patients, whilst retaining a degree of consistency with the original measures. This may be preferable to relying on cancer-specific preference-based measures, which are less useful for comparability across appraisals, and do not themselves always capture these symptoms satisfactorily. It may also be preferable to continually developing new measures to address the shortcomings of existing ones. Such an approach would facilitate a more complete assessment of competing treatments with adverse event profiles that may differ in important ways. It could also reduce the sometimes problematic need for separate adjustment for adverse events in economic models (though some aspects of these events, such as survival outcomes and costs, would still need to be modeled separately from the health-related quality of life data).

Several challenges and research questions remain. While a global treatment-related symptoms item could encapsulate a range of symptoms for a host of current and future treatments (and could even cover symptoms associated with treatments for conditions other than cancer), it is not clear how well such an item would be understood by patients, particularly if they cannot distinguish between the symptoms of their condition and the side effects of treatment. In addition, it is unclear whether general public respondents in a study seeking to obtain utility values for the adapted measure would be able to comprehend valuing a global item that does not refer to specific side effects. Both issues warrant further research prior to adding global treatment-related symptoms items to existing preference-based measures.

It is also unclear what the appropriate recall period would be for treatment-related symptoms, many of which are transient or fluctuate in way that differ from the symptoms of the disease. These issues need investigating in further research in order to assess the case for adding treatment-related symptoms items to an established measure such as the EQ-5D.

Further research is also required into the optimal approach for valuing these additional items. Methods that avoid the need for newly developed value sets bespoke to each new item (and associated measure) are desirable on efficiency grounds and from the perspective of HTA agencies who require a degree of consistency in their methods of assessment and decision-making. Research to date has suggested that adding an item may affect the valuation of the core items of the instrument, so the additional impact on utility may not be simply additive [10]. Further testing of the approach proposed by Yang et al. [53], and alternative methods such as the use of discrete choice experiments to assess preferences for the additional items relative to the core dimensions of the instrument, would be beneficial.

Any adaptation of an existing measure, including development of new treatment-related symptoms items, would require a full assessment of psychometric properties to assess if the adaptation offers an improvement to the status quo. Further, the adapted instrument should also have an impact on associated utility values to offer an improvement. This may not always be the case, as demonstrated by Yang et al. who found that the inclusion of a sleep item did not have a significant impact on utilities derived for EQ-5D-3L health states [42].

The era of immuno-oncology increasingly reveals that current approaches to measuring the impact of cancer treatment-related symptoms on utility values are sub-optimal. This commentary paper has outlined alternative approaches that could be adopted to better capture these impacts for current and future treatments. Further research is needed to test the feasibility of these approaches and assess their impact on decision-making.
